# CZYH Alleviates *β*-Amyloid-Induced Cognitive Impairment and Inflammation Response via Modulation of JNK and NF-*κ*B Pathway in Rats

**DOI:** 10.1155/2019/9546761

**Published:** 2019-11-04

**Authors:** Yuanyuan Deng, Lianzhi Ye, Cheng Yu, Caixia Yin, Jingshan Shi, Qihai Gong

**Affiliations:** ^1^Key Laboratory of Basic Pharmacology of Ministry of Education, Department of Pharmacology, Zunyi Medical University, Zunyi, Guizhou 563000, China; ^2^Joint International Research Laboratory of Ethnomedicine of Ministry of Education, Zunyi Medical University, Zunyi, Guizhou 563000, China

## Abstract

Cu-Zhi-Yi-Hao (CZYH), an empirical formula of traditional Chinese medicine (TCM), has been used for amnesia treatment in clinical practice. However, its underlying pharmacological mechanism has not been fully illuminated. The current study was designed to investigate the neuroprotective effect of CZYH on a *β*-amyloid 25-35- (A*β*_25-35_-) induced learning and memory deficit rat model. CZYH (200, 400, or 800 mg/kg), donepezil (1.0 mg/kg), or distilled water was given to A*β*_25-35_-stimulated animals for 17 days consecutively. The Morris water maze test revealed that CZYH (400 or 800 mg/kg) administration improved the A*β*_25-35_-induced cognitive impairments in rats, and Nissl staining demonstrated that CZYH mitigated the A*β*-caused neuron loss. In addition, CZYH treatment markedly inhibited the activation of microglia as evidenced by a decreased level of IBA-1 and increased YM-1/2 protein expression. The protein expression levels of TNF-*α*, IL-1*β*, and COX-2 were also repressed by CZYH. Besides, CZYH treatment alleviated A*β*-induced I*κ*B-*α* degradation and NF-*κ*B p65 phosphorylation, as well as reduced the JNK phosphorylation level. In conclusion, the present study suggests that CZYH could improve learning and memory abilities and relieve neuron loss in A*β*_25-35_-induced rats, at least partly through inhibition of the neuroinflammatory response via inhibiting the JNK-dependent NF-*κ*B activation, indicating that CZYH might be a promising formula for the treatment of AD.

## 1. Introduction

Alzheimer's disease (AD) is the most prevalent form of neurodegenerative disease [1], with memory loss, cognitive impairment, and behavior disorders as the main clinical manifestations [2], which lead a person unable to execute fundamental bodily functions such as walking and eventually to be bed-bound, seriously decreasing the life quality of patients and increasing the social burden. There are about 44 million people worldwide suffering from AD or a related form of dementia. As the population ages, the number of AD patients will be more than triple to 152 million by 2050 as estimated by epidemiology research. Thus, AD is becoming the most feared disease among elderly people [3].

The pathogenesis of AD is complicated and not fully clarified yet. A*β* hypothesis is predominant in AD research, which suggests that the abnormal release and following accumulation of A*β* are driving causes in the pathogenesis of AD [4]. A*β* is a small peptide generated when the membrane-associated amyloid precursor protein (APP) is cleaved by *β*-secretase (BACE-1) and *γ*-secretase. Meanwhile, A*β* could be degraded by enzymes, such as the insulin-degrading enzyme (IDE) and neprilysin (NEP) [5, 6]. The level of A*β* peptides abnormally increase when the equilibrium between production and clearance is altered, and then the aggregation of A*β* triggers a series of toxic cascade events, including microglial activation, tau phosphorylation, and neuronal degeneration and loss, which lead to memory deficits eventually [7]. Recent evidence indicate AD is also an inflammatory process, as excessive release of A*β* spontaneously aggregates into soluble oligomers, activating the microglia, accompanied by the liberation of inflammatory factors and corresponding inflammation response. These elevated inflammatory factors hamper the resident microglia to remove the A*β*-formed senile plaques, which in turn amplify the inflammation response and A*β* toxicity, speeding up the AD process [8, 9].

Of the more than one hundred drugs that have been tested in the past 2-3 decades, only four have clinical uses; these are cholinesterase inhibitors, including donepezil, rivastigmine, galantamine, and an N-methyl-D-aspartate receptor antagonist memantine. They can alleviate the symptoms of dementia in some patients, but only for some people, and they also bring multiple side effects at the same time due to their single target [10]. Thus, it is imperative to have a combined strategy which takes the body as a whole instead of just one factor.

Traditional Chinese medicine (TCM), based on the idea that everything in our body is connected and considers the overall condition to manage diseases, has been postulated as a promising strategy for AD treatment in Asian countries as it has advantages to keep balance in the human body and then reduce side effects. The Cu-Zhi-Yi-Hao (CZYH) decoction is an empirical formula of TCM in amnesia treatment, composed of eight Chinese herbal medicines including Epimedium, Pueraria, Rehmannia, male Antheraea silkmoths, Salt psyllium, Goji (Wolfberry), Fu Ling (*Poria*), and Coix seed (Table 1). In this decoction, Epimedium and Pueraria are the main components. Epimedium is the leaf part from *Epimedium brevicornum Maxim* and has been used to treat sexual dysfunction, cardiovascular diseases, and dementia for many years. Increasing evidence demonstrated that the two main active compounds of Epimedium, icariin and icariside II, improve cognition in APP/PS1 transgenic mouse models as well as different cognitive deficit rat models induced by streptozocin, A*β*, ibotenic acid, etc. [11–14]. Pueraria itself or its extract has neurotherapeutic effects on cognitive disabled animals [15], Puerarin mitigates A*β*-induced cognitive decline via inhibition of apoptosis response [16], and the ethanol extract of Pueraria acts against trimethyltin-generated learning and memory impairments by repression of acetylcholinesterase (AChE) [17]. A clinical trial of a TCM formula which is composed of Epimedium and another five herbs has displayed a promising therapeutic effect in mild AD patients, and the improving scores were even better than donepezil [18], drawing more attention to use the TCM formula to treat cognitive deficits.

Therefore, in the current study, the effect of the formula CZYH was assessed on an A*β*_25-35_-induced learning and memory impairment rat model. The possible underlying mechanisms were also investigated.

## 2. Materials and Methods

### 2.1. Reagents

Donepezil hydrochloride was bought from Salvage Pharmaceutical Company (Guizhou, China), ground, and dissolved in double-distilled water before use. A*β*_25-35_ was purchased from Sigma-Aldrich (St. Louis, MO, USA) and aggregated by incubation in sterilized normal saline (2 *μ*g/*μ*L) at 37°C for 5 days before injection [11].

### 2.2. Preparation of CZYH Decoction

CZYH is composed of eight constituents (as seen in Table 1), including Epimedium 10 g, Pueraria 10 g, Rehmannia 8 g, male Antheraea silkmoths 8 g, Salt psyllium 4 g, Goji 6 g, Fu Ling 8 g, and Coix seed 8 g. This formula was provided by the Affiliated Hospital of Zunyi Medical University and identified by Professor Jianwen Yang (Zunyi Medical University, Zunyi, China). The mixture was soaked for 1 h with 500 mL distilled water and boiled with gentle heating for 2 h, the dregs were filtered with gauze and the filtrate was collected, then another 375 mL distilled water was added to the dregs and decocted for 2 h, and the above procedure was repeated once, combining the filtrate three times. The extraction was further concentrated, combined, and lyophilized as previously described to get the lyophilized powder [19]. The yield was 25.1% compared with the original crude amount.

### 2.3. Animals

Ninety-eight specific pathogen-free- (SPF-) grade male Sprague Dawley (SD) rats (250-300 g) were provided by the Laboratory Animal Center of the Daping Hospital (license no. SCXK2012-0011) and maintained in a SPF condition (temperature 22 ± 1°C, humidity 58 ± 5%, and 12 h light/12 h dark cycle) in the Animal Center of Key Laboratory of Basic Pharmacology of Ministry of Education, Zunyi Medical University. All animal handling procedures followed the Animal Management Rules of Zunyi Medical University (argument number [2015] 2-043).

### 2.4. Experimental Design

**Figure 1:**
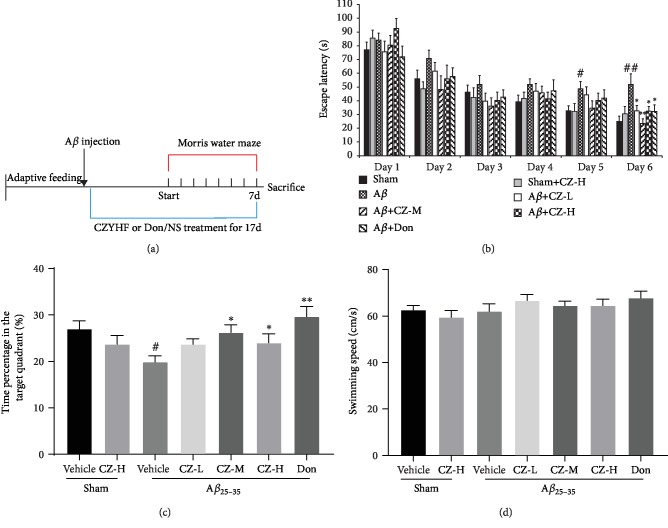
CZYH alleviates learning and memory impairments in A*β*_25-35_-induced rats: (a) experimental design; (b) escape latency; (c) time percentage spent in target quadrant; (d) swimming speed. Data are means ± SEM, *n* = 12‐14. ^#^*P* < 0.05 and ^##^*P* < 0.01 vs. sham; ^∗^*P* < 0.05 and ^∗∗^*P* < 0.01 vs. A*β*_25-35_.

Animals were randomly allocated into seven groups: sham, sham+CZYH-H (800 mg/kg), model (A*β*_25-35_), model+CZYH-L (200 mg/kg), model+CZYH-M (400 mg/kg), model+CZYH-H (800 mg/kg), and model+donepezil (1.0 mg/kg) as the positive drug group (*n* = 14 for each). The sample size was determined by previous experience [20]. The A*β*_25-35_-induced model was conducted following our previous report [11]. Briefly, animals were given a bilateral hippocampal injection of 5 *μ*L A*β*_25-35_ (2 *μ*g/*μ*L) or the same volume NS (sham groups) after being anesthetized with 2% pentobarbital sodium (i.p.). CZYH or donepezil was administered orally daily for 17 days according to the above-described dosage regimen after the animals were woken up from the surgery (Figure 1(a)).

### 2.5. Morris Water Maze

The Morris water maze (MWM) task was applied to estimate the spatial learning and memory function as previously described [12]. The animals were trained and exposed to spatial acquisition trials from day 11 to day 16 after surgery to examine their ability to escape and find the hidden platform. On day 17, a spatial probe trial was carried out to observe the final memory consolidation. All the activities during the trials were automatically recorded and analyzed by TopScan Version 3.00 system.

### 2.6. Nissl Staining

All rats were deeply anesthetized with 2% pentobarbital sodium (intraperitoneally) after the MWM test and perfused with precold 0.01 M phosphate-buffered saline (PBS). The brains were dissociated, and five brains from each group were fixed in 4% paraformaldehyde at 4°C overnight and subsequently embedded in paraffin. Sections (5 *μ*m thick, coronal) of brain tissue were stained with toluidine blue. Blue-purple-stained Nissl bodies of the CA1 and DG regions in the hippocampus were observed and counted to estimate the morphological changes [21].

### 2.7. Western Blotting

The hippocampus tissues were separated and then homogenized in RIPA buffer. The protein concentration of each sample was measured with the BCA method and then heat-blocked and separated as we described before [22]. Immunoblots were probed using primary antibodies against IL-1*β* (1 : 2,000) and COX (1 : 1,000) (obtained from the Abcam company (USA)) and I*κ*B-*α* (1 : 2,000), NF-*κ*B p65 (1 : 1,000), p-NF-*κ*B p65 (1 : 1,000), Akt (1 : 1,000), p-Akt (1 : 1,000), total p38 MAPK (1 : 1,000), p-p38 MAPK (1 : 1,000), JNK (1 : 1,000), and p-JNK (1 : 1,000) (from Cell Signaling Technology (USA)), as well as internal antibodies *β*-actin (1 : 5000) and GAPDH (1 : 5000) (purchased from Beyotime (China)). Subsequently, the membranes were incubated with corresponding secondary antibodies for 90 min at room temperature. The blots were visualized with ECL detection reagents (Beyotime, China) and quantified by One-4.6.7 (Bio-Rad, USA).

### 2.8. Statistics

Statistical analysis was executed using GraphPad Prism 7.04. Values are given as means ± SEM. Repeated-measure analysis of variance (ANOVA) was used for analyzing the MWM data, while one-way ANOVA was performed for the other data; they were followed with Dunnett's test to compare differences among groups.

## 3. Results

### 3.1. CZYH Attenuates A*β*-Induced Cognitive Impairments

In the MWM test, animals displayed declined escape latency across the training period and the A*β*_25-35_-induced rats have longer escape latency than those of the sham group in day 5 and day 6 (Figure 1(b); *P* < 0.05, *P* < 0.01, respectively), indicative of impaired spatial learning ability in the model group. However, CZYH treatment relieved the prolongation of escape latency triggered by A*β*_25-35_ (*F*(6, 85) = 3.316, *P* = 0.0056). The percentage of time spent in the target quadrant was shorter in the A*β*_25-35_-induced group compared with the sham group in the spatial probe test (*F*(6, 85) = 3.25; *P* = 0.0063) (*P* < 0.05), while CZYH 400 mg/kg- or 800 mg/kg-treated rats spent more time in the target quadrant than vehicle-treated animals (*P* < 0.05, *P* < 0.01, respectively). Meanwhile, the swimming speeds of these groups did not exhibit differences (Figure 1(d)), suggesting there is no motor dysfunction between groups. Overall, these results indicated that CZYH alleviated spatial learning and memory deficits induced by A*β*_25-35_.

### 3.2. CZYH Mitigates A*β*-Induced Neuronal Loss

**Figure 2:**
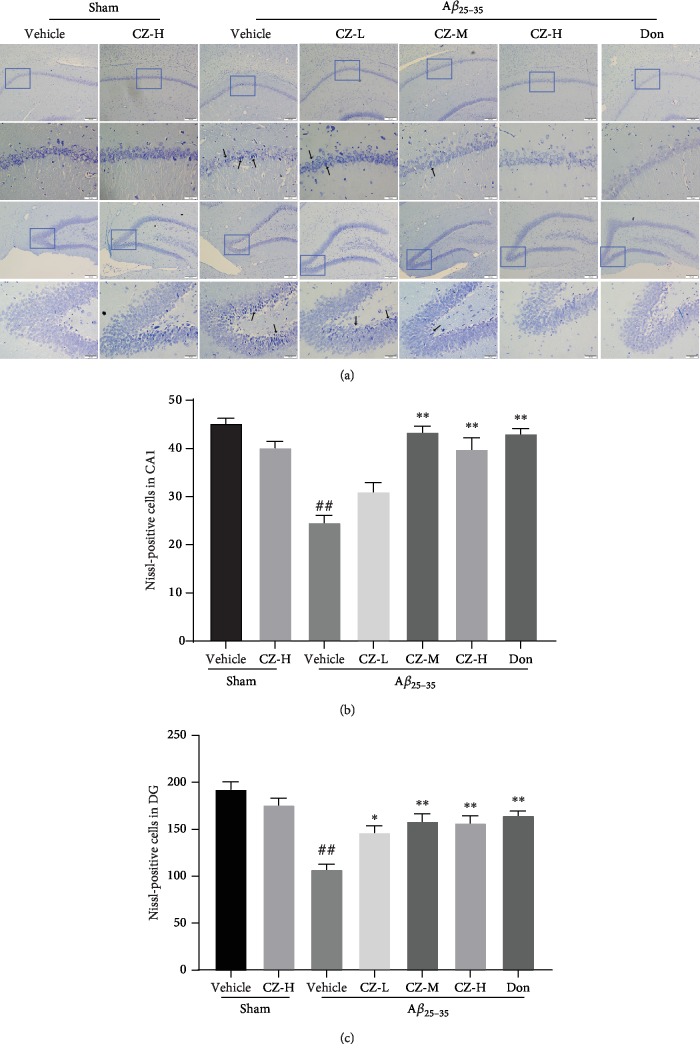
CZYH mitigates A*β*_25-35_-induced neuronal loss in rats. (a) Nissl staining of hippocampus CA1 and DG regions (scale bar: 100 *μ*m and 50 *μ*m). (b, c) Quantitative analysis of Nissl bodies in (b) the CA1 hippocampal region and (c) the DG hippocampal region. Values are means ± SEM, *n* = 5. ^##^*P* < 0.01 vs. sham; ^∗^*P* < 0.05 and ^∗∗^*P* < 0.01 vs. A*β*_25-35_.

The neuronal alterations were assessed by examining the numbers and morphology of neuronal cells in CA1 and DG regions of the hippocampus using Nissl staining. As depicted in Figure 2, highly dense pyramidal layer neurons with uniform and intact structure can be seen in sham groups, whereas lower density with atrophied and disordered neurons were found in the hippocampal CA1 and DG regions of A*β*-induced animals (CA1: *F*(6, 28) = 22.78, *P* < 0.0001; DG: *F*(6, 28) = 14.12, *P* < 0.0001) (*P* < 0.01, *P* < 0.01, respectively). However, the neurons in the CA1 and DG regions revert back to a neat and dense arrangement after CZYH 400 mg/kg or 800 mg/kg treatment. In general, these data suggested that CZYH treatment attenuated neuronal loss induced by A*β*.

### 3.3. CZYH Suppresses A*β*-Induced Microglial Activation and Subsequent Inflammatory Response

**Figure 3:**
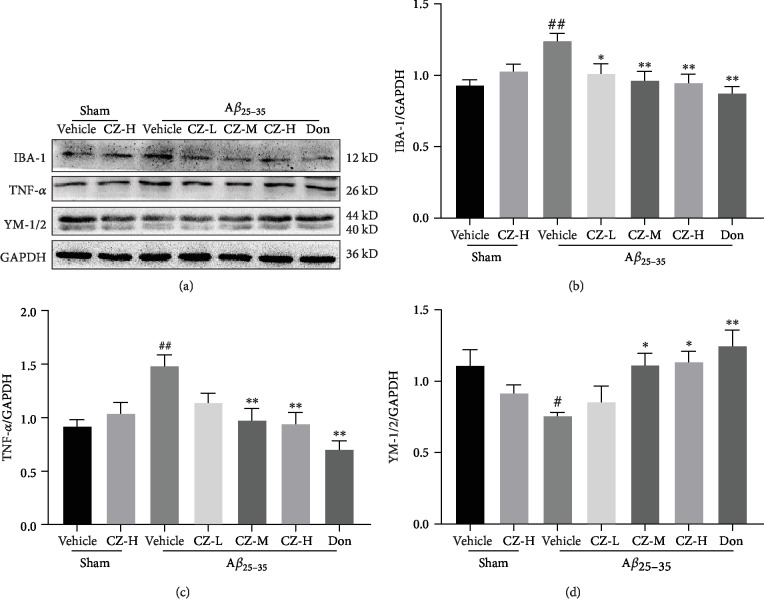
CZYH suppresses A*β*_25-35_-induced proinflammatory microglial activation in the hippocampus of rats. (a) The antibody-reactive band of IBA-1, TNF-*α*, and YM-1/2. (b–d) Quantification of (b) IBA-1, (c) TNF-*α*, and (d) YM-1/2 protein expression levels. Values are means ± SEM, *n* = 5. ^#^*P* < 0.05 and ^##^*P* < 0.01 vs. sham; ^∗^*P* < 0.05 and ^∗∗^*P* < 0.01 vs. A*β*_25-35_.

**Figure 4:**
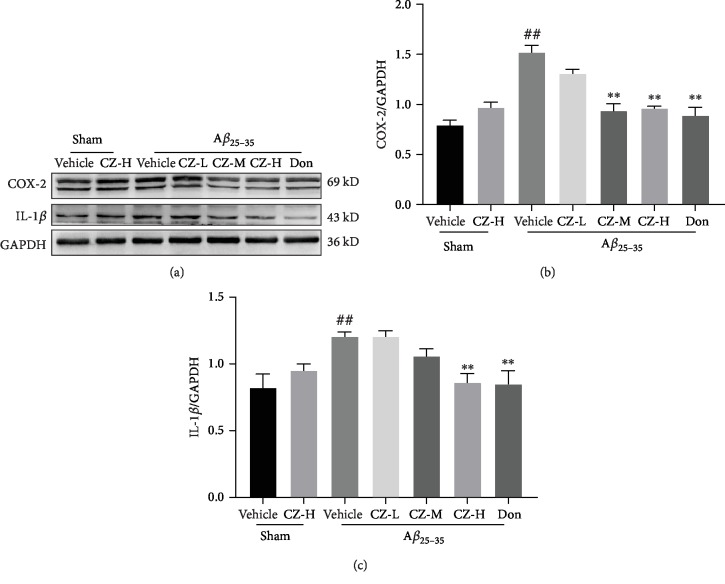
CZYH decreases the expression of inflammatory factors in the hippocampus of A*β*_25-35_-induced rats. (a) The antibody-reactive band of COX-2 and IL-1*β* protein expression. Quantification of (b) COX-2 and (c) IL-1*β* protein expression levels. Values are means ± SEM, *n* = 5. ^#^*P* < 0.05 and ^##^*P* < 0.01 vs. sham; ^∗^*P* < 0.05 and ^∗∗^*P* < 0.01 vs. A*β*_25-35_.

Since the inflammatory process is involved in A*β* toxicity, Western blotting was used to determine the activation of microglia and the expression of inflammatory factors. The expression of microglial activation marker IBA-1 was enhanced in the hippocampi of A*β*-treated rats compared to those of the sham group (*F*(6, 28) = 4.702, *P* = 0.0020) (*P* < 0.01, Figure 3), suggesting microglial activation and neuroinflammation. Meanwhile, the level of the proinflammatory microglial (M1) phenotype marker TNF-*α* was increased (*F*(6, 28) = 6.462, *P* = 0.0002) (*P* < 0.01), and the anti-inflammatory microglial (M2) marker YM-1/2 was decreased notably (*F*(6, 28) = 4.225, *P* = 0.0038) (*P* < 0.05). However, CZYH treatment reduced the expression of both IBA-1 and TNF-*α* and upregulated the levels of YM-1/2, pointing that treatment with CZYH represses microglial M1 activation and promotes microglia to polarize to the neuroprotective M2 phenotype. Additionally, the inflammatory factors including COX-2 and IL-1*β* expression were increased in A*β*-injected animals (*F*(6, 28) = 19.85, *P* < 0.0001; IL-1*β*: *F*(6, 28) = 5.735, *P* = 0.0005) (*P* < 0.01, *P* < 0.01, respectively) and decreased after CZYH or donepezil treatment (Figure 4), further confirming CZYH could subside inflammatory response induced by A*β*.

### 3.4. CZYH Lessens A*β*-Induced I*κ*B-*α* Degradation and NF-*κ*B p65 Phosphorylation

**Figure 5:**
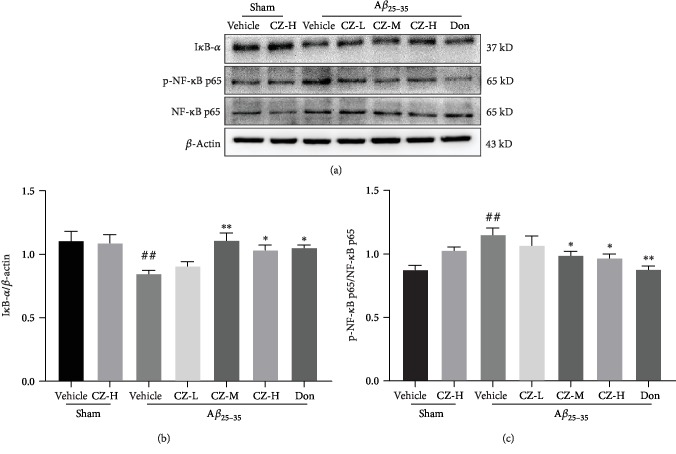
CZYH represses I*κ*B-*α* degradation and NF-*κ*B p65 phosphorylation induced by A*β*_25-35_ in the hippocampus of rats. (a) The antibody-reactive band of I*κ*B-*α* and p-NF-*κ*B p65 protein expression. (b, c) Statistics of (b) I*κ*B-*α* and (c) p-NF-*κ*B p65 levels. Values are means ± SEM, *n* = 5. ^##^*P* < 0.01 vs. sham; ^∗^*P* < 0.05 and ^∗∗^*P* < 0.01 vs. A*β*_25-35_.

As NF-*κ*B is a pivotal transcription factor in regulating inflammation, immune responses, and cell survival [23], the phosphorylation of NF-*κ*B p65 and expression of its major inhibitor protein I*κ*B-*α* were further scrutinized. As illustrated in Figure 5, the I*κ*B-*α* level was markedly declined after A*β* injection (*F*(6, 28) = 4.338, *P* = 0.0032) (*P* < 0.01) and was escalated following CZYH 400 mg/kg or 800 mg/kg treatment (*P* < 0.01, *P* < 0.05). Conversely, the phosphorylation level of NF-*κ*B p65 was increased in A*β*-induced rats compared to the level of the sham group (*F*(6, 28) = 5.499, *P* = 0.0007) (*P* < 0.01), and the elevated NF-*κ*B p65 phosphorylation was efficiently repressed by CZYH 800 mg/kg and donepezil administration.

### 3.5. CZYH Restrains A*β*-Induced JNK Phosphorylation

**Figure 6:**
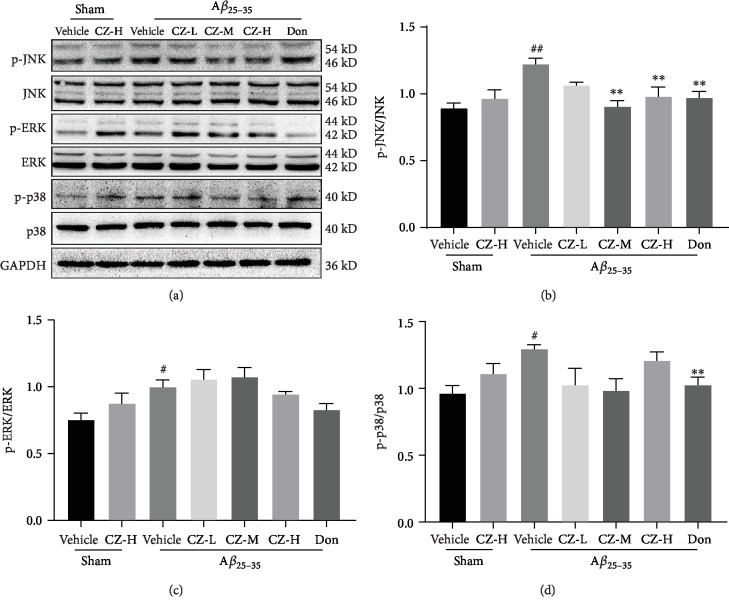
CZYH attenuates JNK phosphorylation induced by A*β*_25-35_ in the hippocampus of rats. (a) Representative bands of p-JNK, JNK, p-ERK, ERK, p-p38, and p38. (b–d) Statistics of (b) JNK, (c) ERK, and (d) p38 phosphorylation. Values are means ± SEM, *n* = 5 for JNK and ERK and *n* = 3 for p38. ^#^*P* < 0.05 and ^##^*P* < 0.01 vs. sham; ^∗^*P* < 0.05 and ^∗∗^*P* < 0.01 vs. A*β*_25-35_.

Mitogen-activated protein kinases (MAPKs) are a family of kinases involved in mediating intracellular signaling including inflammation. In order to clarify the mechanisms underlying the protection effects of CZYH against A*β*-induced cognitive deficits, Western blot was utilized to examine the activation of MAPK family members: c-Jun N-terminal kinase (JNK), extracellular signal-related kinase (ERK), and p38 MAPK. All the three MAPK members' (JNK, ERK, and p38) phosphorylation levels were upregulated after A*β* stimulation (*P* < 0.01, *P* < 0.05, and *P* < 0.05, respectively), while the upraised phosphorylation level of JNK was reduced after CZYH or donepezil treatment (Figure 6). However, the elevated level of p-ERK can only be suppressed by donepezil instead of different doses of CZYH. Broadly speaking, CZYH treatment could attenuate the A*β*-induced phosphorylation of JNK, but not ERK and p38.

## 4. Discussion

Although AD is a multifactorial disease with a not clearly understood pathogenesis, multiple studies have shown that A*β* aggregation and deposition is the driving cause of AD. The intrahippocampal injection of a fragment of A*β* can mimic pathological and behavioral features of AD; A*β*-induced animal models have been widely used to evaluate pharmacological therapies for AD. Consistent with previous studies [11], we confirmed that a single injection of A*β*_25-35_ into the rat's hippocampus leads to learning and memory impairments and neuronal damage in CA1 and DG subfields of the hippocampus. Importantly, the above deficits could be effectively prevented by CZYH 400 or 800 mg/kg treatment. It is worth noting that, even if 200 mg/kg CZYH administration was able to reduce the abnormally elevated escape latency for A*β*_25-35_-induced rats in the MWM test, it has insignificant effect on improving spatial memory (Figure 1(c)) and neuron loss (Figure 2), suggesting that the effective dose of CZYH is above 200 mg/kg. Moreover, the effect of CZYH was not in a dose-dependent manner; it was speculated that the disparity between doses in the current study is too close to cause a significant difference in effect.

Neuroinflammation is linked to the mechanisms of A*β*-induced toxicity and AD pathogenesis. Excessive level of A*β* can activate microglial cells to release inflammatory cytokines, including COX-2, IL-1*β*, and TNF-*α* [24]. These elevated proinflammatory factors impede resident microglia to phagocytosis A*β*, which in turn aggravate the inflammatory response and AD development [25]. There are two phenotypes of activated microglia; one is the classic activated microglia (also known as the proinflammatory state, M1) which has been characterized by secreting inflammatory cytokines; another one is the alternative activated state (anti-inflammatory state, M2) and is regarded to be beneficial as it may delay the progression of the disease. The present study revealed that the expression of M1 markers was significantly enhanced after A*β*_25-35_ stimulation, whereas YM-1/2 expression (M2 marker) was decreased, consistent with the recent study which showed A*β*_1-42_ can activate N9 microglia into the M1 state [26], and the current findings firstly reveal that A*β*_25-35_ could induce hippocampus microglial M1 activation *in vivo*. In addition, elevated levels of COX-2, IL-1*β*, and TNF-*α* were also detected in A*β*_25-35_-induced animals, implying that exogenous injection of A*β*_25-35_ stimulates microglia to M1 polarization which then secrete proinflammatory factors, forming chronic neuroinflammation conditions in the brain, eventually resulting in neuronal loss and associated cognitive decline. Consistent with the studies about donepezil [6, 27], we also find that donepezil inhibited M1 activation and promotes M2 polarization in microglia and then suppressed the release of inflammatory cytokines. It is of note that similar anti-inflammatory effects have been seen in CZYH as evidenced by the reduced level of IBA-1 and the elevated level of YM-1/2, and decreased expression of inflammatory factors was also observed in CZYH-treated groups.

NF-*κ*B, a well-characterized transcription factor, has an important role in regulating the expressions of several proinflammatory cytokines. NF-*κ*B was found activated in microglial cells in the brains of AD patients [28], and it has been demonstrated that NF-*κ*B is involved in the neuroinflammatory response induced by A*β* [21, 29]. NF-*κ*B is retained as inactive by binding with I*κ*B proteins in physiological conditions. Upon stimulation, the I*κ*B kinase is phosphorylated and degraded to empower NF-*κ*B nuclear translocation, subsequently initiating the transcription of downstream genes, including inflammatory cytokines. The current study confirmed that A*β*_25-35_ mediated the degradation of I*κ*B-*α* and the subsequent NF-*κ*B p65 phosphorylation, leading to the release of the microglia-derived proinflammatory factors. However, the activation of NF-*κ*B p65/I*κ*B-*α* could be ameliorated by CZYH treatment, which is consistent with the observations that *Epimedium* and a prescription contain *Epimedium* modulate neuroinflammatory response via suppressing of the NF-*κ*B pathway [30–32].

MAPKs are a family of protein kinases that are specific to the amino acids serine and threonine. Increasing lines of evidence provided strong support that MAPK regulates the activities of several transcription factors, including NF-*κ*B [33, 34]. There are three major subgroups of MAPK: JNK, ERK, and p38, with distinct roles in regulating cell functions. ERK is involved in growth factor signaling, including cell proliferation, cell cycle progression, cell division, and cell differentiation [35], while the JNK and p38 signaling pathways are responsive to stress stimuli, such as cytokines, heat shock, and radiation [36, 37]. This study found that A*β*_25-35_ injection elicited the phosphorylation of ERK and p38, as well as JNK, in the hippocampus, adding more evidence to support and extend the notion that neuroinflammatory signals stimulated by A*β* in microglia via phosphorylation of MAPK initiate I*κ*B-*α* degradation following NF-*κ*B activation, which immediately mobilizes the transcription of inflammatory molecules. In addition, we observed that CZYH reduced the JNK kinase phosphorylation activity in the hippocampus of A*β*_25-35_-induced rats but has no significant effect on the phosphorylation of p38 or ERK. However, donepezil is able to alleviate both the JNK phosphorylation and the p38 phosphorylation, which is coincident with a previous report [38]. These results suggest that CZYH mediated decrease in JNK phosphorylation and then inhibited the downstream activation of NF-*κ*B, accounting for the suppression of neuroinflammatory response in A*β*_25-35_-induced rats.

In conclusion, this study demonstrated that CZYH attenuates cognitive impairments and neuron loss induced by A*β*_25-35_, owing to its anti-inflammatory effects via the suppression of JNK phosphorylation and the blockade of the NF-*κ*B/I*κ*B-*α* pathway. Therefore, our findings suggest that Chinese herbal formula CZYH could be beneficial and effective for AD treatment.

## Figures and Tables

**Table 1:** Composition of Cu-Zhi-Yi-Hao.

Common name	Latin name	Quantity (g)
Epimedium	*Epimedium brevicornum Maxim*	10
Pueraria	*Pueraria edulis Pampan*	10
Rehmannia	*Rehmanniae Radix*	8
Male Antheraea silkmoths	*Bombycidae*	8
Salt psyllium	*Semen Plantaginis*	4
Goji	*Lycii Fructus*	6
Fu Ling	*Poria*	8
Coix seed	*Semen Coicis*	8

## Data Availability

The data used to support the findings of this study are available from the corresponding author upon request.

## Abbreviations

AD: Alzheimer's disease
A*β*: Beta-amyloid
ANOVA: Analysis of variance
APP: Beta-amyloid precursor protein
BACE1: Beta-site APP-cleaving enzyme 1
BCA: Bicinchoninic acid
COX-2: Cyclooxygenase-2
CZYH: Cu-Zhi-Yi-Hao
ERK: Extracellular signal-related kinase
IDE: Insulin-degrading enzyme
JNK: c-Jun N-terminal kinase
MAPK: Mitogen-activated protein kinase
MWM: Morris water maze
NF-*κ*B: Nuclear factor *κ*B
PVDF: Polyvinylidene fluoride
SD: Sprague Dawley
SEM: Standard error of the mean
SPF: Specific pathogen free
TCM: Traditional Chinese medicine
TNF-*α*: Tumor necrosis factor-*α*.
